# A Review of Wine Authentication Using Spectroscopic Approaches in Combination with Chemometrics

**DOI:** 10.3390/molecules26144334

**Published:** 2021-07-17

**Authors:** Ranaweera K. R. Ranaweera, Dimitra L. Capone, Susan E. P. Bastian, Daniel Cozzolino, David W. Jeffery

**Affiliations:** 1 Department of Wine Science and Waite Research Institute, The University of Adelaide, PMB 1, Glen Osmond, SA 5064, Australia; ruchira.ranaweera01@adelaide.edu.au (R.K.R.R.); dimitra.capone@adelaide.edu.au (D.L.C.); sue.bastian@adelaide.edu.au (S.E.P.B.); 2 Australian Research Council Training Centre for Innovative Wine Production, The University of Adelaide, PMB 1, Glen Osmond, SA 5064, Australia; 3 Queensland Alliance for Agriculture and Food Innovation (QAAFI), The University of Queensland, Hartley Teakle Building, Brisbane, QLD 4072, Australia; d.cozzolino@uq.edu.au

**Keywords:** authenticity, multivariate analysis, wine fingerprinting, spectral data, machine learning

## Abstract

In a global context where trading of wines involves considerable economic value, the requirement to guarantee wine authenticity can never be underestimated. With the ever-increasing advancements in analytical platforms, research into spectroscopic methods is thriving as they offer a powerful tool for rapid wine authentication. In particular, spectroscopic techniques have been identified as a user-friendly and economical alternative to traditional analyses involving more complex instrumentation that may not readily be deployable in an industry setting. Chemometrics plays an indispensable role in the interpretation and modelling of spectral data and is frequently used in conjunction with spectroscopy for sample classification. Considering the variety of available techniques under the banner of spectroscopy, this review aims to provide an update on the most popular spectroscopic approaches and chemometric data analysis procedures that are applicable to wine authentication.

## 1. Introduction

Wine is a historic alcoholic beverage that has evolved to be of high commercial importance. It can be identified as a luxurious commodity and is produced and consumed in many countries around the world. Wine consists of innumerable compounds spanning various concentration ranges, many of which are essential to its evolution and quality, as well as for human health benefits in the case of red wine [1]. In general, the composition of red wine can be broadly represented on a w/w basis as 86% water, 11% ethanol, and 3% for the remainder, which includes glycerol, sugars, polyols, phenols, minerals, organic acids, and volatile compounds [2]. The composition of wine mainly depends on certain factors that define the wine’s identity, including grape variety, geographical origin, the biophysical environment of the vineyard, vintage conditions, and winemaking inputs [3]. Different types of fraud related to those factors have been encountered in wine over the years, including counterfeiting of labels and brands, adulteration through the use of unauthorised additives or practices, and substitution based on grape variety or region of origin [4]. Therefore, to confirm the genuineness of wine and protect its value, analytical techniques need to be applied to explore the chemical constituents of wine that aid in the development of models for authenticity.

**Figure 1:**
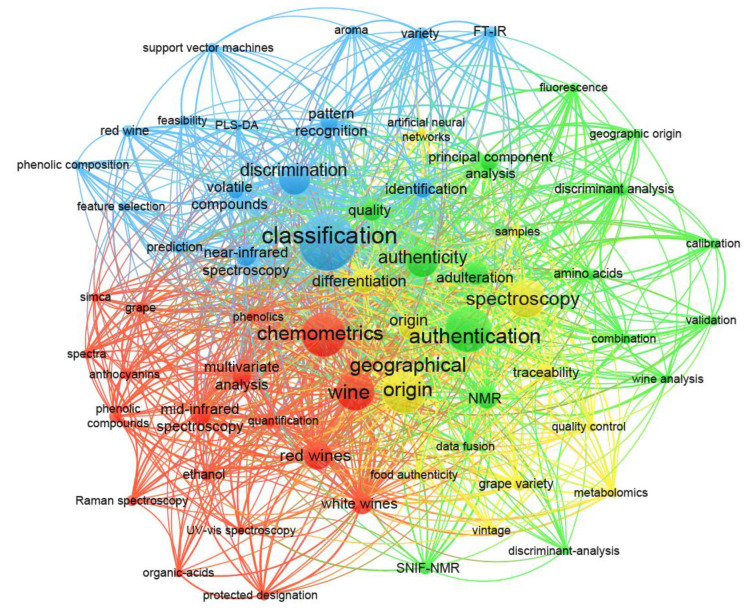
Bibliometric map of wine science-related research visualised from 222 publications (from 1990 to 2021) recovered from Web of Science Core Collection using ‘wine authentication’ and ‘spectroscopy’ as keywords. Literature analysis and figure construction were facilitated with VOSviewer [12]. Different colours are used to define the clusters that terms belong to. The bibliometric relationship between terms is indicated using curved lines and the relative size of the words reflects the number of publications in which the terms occurred.

Classical techniques such as gas chromatography-mass spectrometry and high-performance liquid chromatography are advancing continuously, facilitating wine analysis with high sensitivity [5]. Considering the applicability in an industrial setting, however, aspects such as rapidity, user-friendliness, and cost-effectiveness have become of paramount importance in recent times [6]. Spectroscopic techniques provide a great solution due to their relative simplicity, speed of analysis, simple sample preparation, and environmental friendliness [7], and have been well-utilised for different wine and grape research studies, such as for targeted and non-targeted chemical analyses [8], prediction of sensory attributes [9], and wine authentication [10,11]. A snapshot of selected research outcomes identified from the Web of Science Core Collection over the past three decades using ‘wine authentication’ and ‘spectroscopy’ as the search keywords is visualised in Figure 1 to provide some understanding of the trends in the literature. Aside from those specific keywords, the terms classification, chemometrics, and geographical origin also feature prominently and are variously linked upon closer inspection to a range of terms associated with spectroscopic (e.g., near-infrared, mid-infrared, NMR, UV–visible, Raman, fluorescence) and chemometric (e.g., partial least squares discriminant analysis, feature selection, support vector machines, artificial neural networks, principal component analysis, discriminant analysis, data fusion, pattern recognition) techniques.

Among the different spectroscopic methods that are available, techniques such as nuclear magnetic resonance (NMR), near-infrared (NIR), mid-infrared (MIR), Raman, and fluorescence have been prominent in past research studies. Moreover, it is clear from Figure 1 that chemometric techniques (i.e., multivariate data analyses) have been an integral part of these spectroscopic techniques to draw meaningful conclusions regarding sample classification and differentiation. Taking these aspects together, this review emphasises the application of spectroscopic techniques and chemometrics to authenticity in the field of wine research using examples from the past 15 years. The strengths and weaknesses of different spectroscopic methods for wine authentication are presented and various chemometric methods applied to address specific requirements in classification are discussed. Finally, future trends and directions for wine authentication with spectroscopic approaches have been identified.

## 2. Spectroscopic Techniques Applied in Wine Authentication

Wine authentication verifies that the label description is in compliance with the content of the package through an analytical process [13], which can be carried out through targeted or non-targeted methods. In targeted analyses, variations of a specific marker compound or certain metabolites are considered for differentiation of samples, whereas in non-targeted analyses, a chemical ‘fingerprint’ of the sample is obtained and similarities/differences in fingerprint are used for classification with the aid of chemometrics [14]. Spectroscopic techniques are frequently utilised for non-targeted wine fingerprinting.

**Figure 2:**
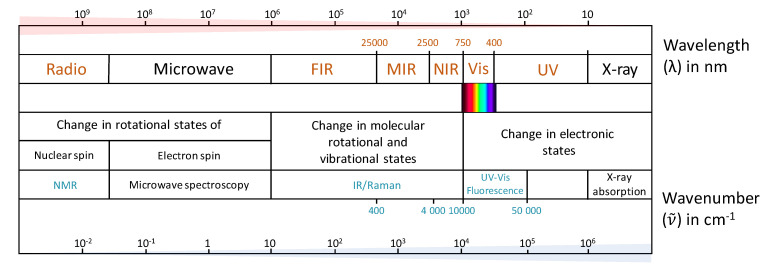
The electromagnetic spectrum and its relevance to different spectroscopic methods. FIR, far-infrared; MIR, mid-infrared; NIR, near-infrared; Vis, visible; UV, ultraviolet. Techniques in this review that are applied for wine authentication are indicated in light blue font. Conceptualised from [15].

In spectroscopic analysis, chemical and physical (structural) information within samples is exploited according to the interaction of atoms and molecules with electromagnetic radiation (Figure 2), which related to the wavelength or frequency spectrum of either absorbed or emitted energy [14]. For instance, ultraviolet-visible (UV–Vis) absorption and fluorescence spectroscopy is based on changes that occur in electronic states. In another way, infrared (IR) and Raman spectroscopic techniques are based on vibrational variations in the molecules. Moving further along the electromagnetic spectrum to longer wavelengths past the microwave region, NMR involves changes in rotational state, with nuclear spin being affected within the radiofrequency range. Data obtained from these methods typically needs to be analysed through multivariate techniques to obtain useful information hidden in the spectra. For authentication purposes, data can then be further classified using statistical approaches as outlined in Section 3. Firstly though, the main spectroscopic techniques applied for wine classification (as identified from Figure 1) are reviewed, which necessarily involves some mention of chemometrics.

### 2.1. UV–Vis Spectroscopy

UV–Vis spectroscopy is a fast, low-cost, and reliable analytical method that has been used in the analysis of wine for many decades [16]. Spectra recorded at UV and visible wavelengths (typically 190–800 nm, Figure 2) provide information about compounds in wine containing a chromophore, such as hydroxybenzoic (280 nm) and hydroxycinnamic (320 nm) acids, flavan-3-ols (280 nm), flavonols (370 nm), and anthocyanin glucosides (520 nm) [17]. As summarised in Table 1, UV–Vis spectroscopy has been applied in wine discrimination according to the region of origin [18,19], grape variety and ageing process [20,21]. Although the specific chemical markers are not necessarily identified, as a non-targeted method combined with appropriate chemometric techniques such as linear discriminant analysis (LDA) and partial least squares discriminant analysis (PLS-DA), Azcarate et al. were able to correctly classify Argentinian Sauvignon blanc wine samples with 100% accuracy according to their geographical origin [19]. In their study, Philippidis et al. achieved 97.5% correct classification of grape variety and showed that the latent variables resulting from orthogonal projections to latent structures-discriminant analysis (OPLS-DA) could be related to the absorption of aromatic compounds such as phenolic acids and flavonols [21]. In comparison to other spectroscopic methods, however, UV–Vis spectroscopy provides a limited number of spectral features; therefore, it could be used as a screening approach with more sophisticated techniques being implemented for further analysis. In addition, the combination of other spectroscopic methods like IR and fluorescence with UV–Vis spectroscopy can improve the accuracy of classification models used for authentication by fusion of the datasets [22,23].

### 2.2. IR Spectroscopy

IR spectroscopy has been used in wine analysis for several decades [24] and has become the most frequently applied spectroscopic technique in comparison to other methods [25]. It is a user-friendly and rapid technique that provides information on many components in a wine matrix, and can be used for determination of parameters such as alcohol content, pH, volatile acidity, organic acids, reducing sugars, and polyphenols [26]. Two main IR-based techniques are applied according to the range in the spectral region: near-infrared (NIR) from 14,000 to 4000 cm^−1^ and mid-infrared (MIR) from approximately 4000 to 400 cm^−1^ (Figure 2). NIR spectra contain less intense bands than MIR and it is difficult to assign chemical groups specifically with NIR due to overlapping signals with water and ethanol around 1950 nm. In MIR, there is a ‘fingerprint region’ (1500–400 cm^−1^) that includes unique absorption patterns of compounds such as phenolics that are mainly applied for discrimination purposes, and signals associated with various functional groups can be assigned, such as C=O related to organic acids at 1700 cm^−1^, and combinations of C–H vibrations and overtones related to ethanol and sugars at around 2300–2100 cm^−1^ [27]. Applicability of IR methods to wine analysis increased with the introduction of techniques such as Fourier transform (FT), which has improved data collection speed and reproducibility [28], and the application of attenuated total reflectance (ATR), which simplifies the sample handling process and is advantageous in routine analysis [29]. Classification of wine with IR has often been complemented by the use of UV and/or visible spectroscopy to enhance the accuracy of the classification [30]. Table 2 includes some examples of the application of IR spectroscopy (with or without UV–Vis) to wine authentication, along with the spectral region and classification method used.

In another study, Bevin et al. discriminated Australian red wine (Cabernet Sauvignon, Shiraz and Merlot) and white wine (Chardonnay, Riesling, Sauvignon blanc and Viognier) according to grape variety with 96% and 94% accuracy, respectively, using LDA with MIR spectra [26]. Although subtle variation in wine composition contributed to these varietal discriminations, MIR signals are highly sensitive to temperature and pH, which needs to be considered in the application. For geographical authentication, Cozzolino et al. combined NIR and MIR techniques for Sauvignon blanc wines from Australia and New Zealand, achieving an overall 93% correct classification with PLS-DA, which was higher than for the individual IR techniques or for UV–Vis [23]. Similarly, the feasibility of differentiating subzones within a denomination of origin (DO) has been evaluated by Martelo-Vidal et al., who achieved their highest overall correct classification of 86% with LDA in comparison to soft independent modelling of class analogy (SIMCA, 56%) and support vector machine (SVM, 84%) for combined UV–Vis and NIR spectra [30]. Hu et al. applied MIR and NIR to classify Cabernet Sauvignon wines with SIMCA and correctly classified Australian, Chilean, and Chinese wines with 97%, 97%, and 92% accuracy, respectively [31]. Although these works yielded an accuracy of > 90% for classification, IR spectroscopy has limitations in quantitative analysis when measuring low abundance components (<0.5 g L^−1^) [32].

### 2.3. Raman Spectroscopy

In comparison to other spectroscopic techniques, Raman spectroscopy has not been exploited much for wine analysis until recently [33]. This spectroscopic method involves detecting the inelastic scattered light emitted from molecular vibrations of a sample, approximately in the range 200–3600 nm (Figure 2). The Raman effect produces a weak signal, but the development of optimised detection capability provides the opportunity to obtain rich information regarding the chemical composition and dynamics of the sample [34]. There are two different regions in Raman spectroscopy, with Stokes Raman scattering having more dominant ethanol, sucrose and water peaks, and anti-Stokes Raman scattering from minor components such as aromatic compounds, including various phenolics, which can be more applicable to wine discrimination [35]. Indeed, for analysis of water dominant samples such as wine, Raman spectroscopy has an advantage over IR techniques because of the relatively weak signals from water molecules in the vibrational fingerprint range [36]. Two types of Raman technique are applied in food analysis: FT-Raman spectroscopy and surface-enhanced Raman spectroscopy (SERS). Both of these methods have been developed for the purpose of wine authentication, as shown in Table 3.

The effectiveness of FT-Raman was shown in the work of Magdas et al., who discriminated white wine according to variety (Sauvignon, Riesling, Chardonnay, Pinot Gris), geographical origin (Romania and France), and vintage using LDA, achieving overall correct classification of 84%, 100%, and 95%, respectively [34,37]. In another study, Magdas and colleagues applied SERS to discriminate among white wines and compared it with FT-Raman, identifying a few common marker compounds between the techniques, such as ferulic and sinapic acids that resulted in differences among the wines. SERS was able to enhance the signals of more minor compounds such as caffeic acid, *p*-coumaric acid and resveratrol [35]. Applying the same SERS approach, Zanuttin et al. discriminated wines according to variety and producer with SIMCA, deriving an overall correct classification of 87%. Moreover, they identified major metabolites such as purines, carboxylic acids and glutathione that can be assigned to specific bands responsible for discrimination of wine [38]. The advantage of SERS over FT-Raman is the selectivity afforded by specific molecules being adsorbed to metal nanostructures (mainly noble metals), which enhances the intensity of Raman signals in SERS [39]. Complexity arises with the sample preparation step, however, as it is necessary to prepare a colloidal dispersion of Ag nanoparticles to add to the sample, which can be a disadvantage. Raman spectroscopy requires spectral pre-processing such as multiplicative scatter correction (discussed in Section 3) to avoid the effect of fluorescence that can obscure Raman scattering, especially when analysing wine [40].

### 2.4. Fluorescence Spectroscopy

Fluorescence spectroscopy has been deemed as a useful tool in wine authentication for some time and its application has been enhanced recently with improvements in the chemometric analysis [41]. Because of the high sensitivity, selectivity, and rapidity of the technique, fluorescence spectroscopy has an advantage as an analytical platform [42]. It is based on the emission of longer wavelength light from a substance after absorption of energy in the UV or visible range (as with UV–Vis spectroscopy, Figure 2). Fluorescence typically occurs for aromatic molecules and can be well applied to wine analysis, with common fluorophores being a variety of phenolic compounds, vitamins, and aromatic amino acids [43]. According to the fluorophoric molecular and macromolecular constituents in the sample, a three-dimensional excitation-emission matrix (EEM) recorded over multiple excitation and emission wavelengths can be obtained and considered as the ‘molecular fingerprint’ of the sample [44]. Therefore, this approach in combination with chemometrics can be utilised for authentication of wine. When undertaking spectrofluorometric analysis, it is important to apply corrections for Rayleigh masking, Raman scattering, and inner filter effects (IFE), as well as to maintain proper pH and temperature to avoid the consequence of quenching, which can affect the fluorescence intensity. Several types of fluorescence methods can be applied to wine analysis according to the manner of obtaining the spectrum (i.e., total luminescence spectroscopy yielding an EEM or synchronous fluorescence spectroscopy) and by the geometry of sample illumination (i.e., right-angle for diluted samples or front-face for bulk liquids or solids) [45]. Fluorescence spectroscopy has been applied in several studies recently, in combination with chemometric techniques, for discrimination of wine according to geographical origin or variety (Table 4).

Sádecká and Jakubíková applied synchronous fluorescence spectroscopy to discriminate white wine according to variety (Furmint, Lipovina, and Muscat blanc) using LDA, achieving an overall rate of 100% correct classification in validation and 93% for prediction [46]. Using total luminescence spectroscopy for authentication, Suciu et al. classified white wine according to geographical origin (Romania and France) and variety (Chardonnay, Pinot Gris, Riesling and Sauvignon), obtaining correct classification rates of 98% and 97.1%, respectively, by applying parallel factor analysis (PARAFAC) and SIMCA algorithms [47]. Based on an absorbance-transmission and fluorescence excitation-emission matrix (A-TEEM) approach that also uses right-angle geometry with total fluorescence spectroscopy, Ranaweera et al. classified Cabernet Sauvignon wines according to geographical origin with 100% accuracy using EEM data and a machine learning algorithm known as extreme gradient boosting discriminant analysis (XGBDA) [11]. This was contrasted with SVM as an alternative machine learning technique, which gave 85% correct classification according to region. In a subsequent study, those authors used A-TEEM in conjunction with XGBDA to classify over 200 commercially produced but unreleased Australian red wines by origin and variety with 99.7% and 100% accuracy, respectively [48]. This method involved multi-block data analysis of EEM and absorbance datasets, as well as PARAFAC to extract components according to the major fluorophores differentiating the wines.

### 2.5. NMR Spectroscopy

Among the most mature forms of spectroscopy for food and beverage classification, NMR has been applied to wine authentication for decades. Initially, site-specific natural isotopic fractionation NMR (SNIF-NMR) spectroscopy was proposed as a tool for detecting the biochemical origin of ethanol according to the natural distribution of deuterium [49], which can reveal the unauthorised use of chaptalisation (sugar addition) in winemaking, for example [50]. NMR spectroscopy can be applied for qualitative analysis to determine molecular structures and for compositional profiling of a sample [51], as well as for quantitative analysis of analytes such as amino acids, alcohols, sugars, carboxylic acids and their derivatives, and phenolic compounds [50]. NMR can be based on acquisition of ^1^H, ^2^H, or ^13^C spectra; for wine authentication, ^1^H NMR spectroscopy is most advantageous as data acquisition is fast and highly reproducible compared to other techniques [33]. Moreover, NMR with advancements such as automation of analysis has been introduced commercially and adapted to wine authentication (e.g., Bruker’s WineScreener^TM^) [50]. Using the possibilities of NMR spectroscopy, different aspects of wine authenticity have been addressed (Table 5).

Using the entire ^1^H NMR spectrum as a fingerprint in conjunction with LDA, Godelmann et al. classified German wines from five regions according to geography, variety and vintage with overall correct classifications of 89% (geographical), 95% (varietal), and 96–97% (vintage) [52]. ^1^H NMR metabolomic data has also been applied for quantification of a range of metabolites including sugars, amino acids, organic acids, alcohols, and phenolic compounds, which were used for wine discrimination as a function of terroir (encompassing biophysical and cultural factors of the production region) and cultivar [55]. Moreover, Alexandra et al. explored the possibility of combining untargeted ^1^H NMR analysis with targeted peptide based sensing arrays to classify Pinot noir wines on the basis of characteristic metabolic signatures associated with variations in terroir [56]. Other recent studies have also used ^1^H NMR as a nontargeted method for authentication. Fan et al. subjected 99 red and 71 white wines from China to NMR analysis, subsequently using segment-wise peak alignment followed by PCA and LDA for separating red and white wine samples as well as different varieties [53]. Similarly, Mascellani et al. used NMR to classify over 900 Czech wines according to type (based on colour and residual sweetness) and variety using a random forest (RF) machine learning algorithm [54]. Correct classification according to wine type was 92% or more for white wine styles (dry and medium dry, medium, sweet) and > 99% for dry red, but the chosen model was unable to provide correct classification for all varieties, with some varieties such as Sauvignon blanc, Pinot Gris, Pinot blanc, and Pálava being below 50% accuracy. Overall, NMR is shown to be an effective technique for authentication with rapid determination of range of metabolites, even if it has become the most expensive spectroscopic approach [33].

In the selection of techniques, it is important to consider the various merits and characteristics of the approaches and to evaluate these according to the question to be addressed. Thus, despite the potential challenges, each of the reviewed methods prevail due to their usability in wine authentication. A summary of the techniques including perceived advantages and disadvantages is presented in Table 6.

## 3. Application of Chemometrics for Modelling with Spectroscopic Data

**Figure 3:**
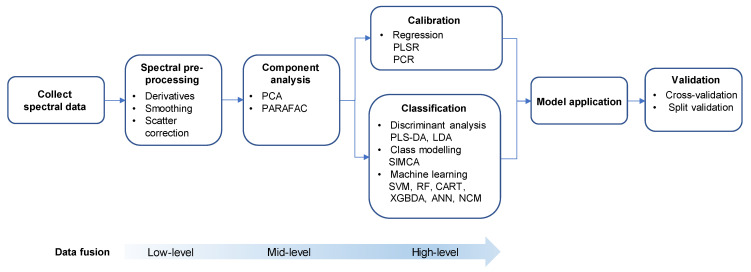
Schematic of the steps involved with chemometric analysis of spectroscopic data. Stages where data fusion can be applied depending on the extent of processing are also presented in the diagram.

Spectroscopic methods rapidly produce an abundance of variables (peak intensities and wavelengths) that need to be dealt with. Therefore, to analyse these high dimensional sets of ‘big data’, integration with appropriate multivariate statistical analysis methods (i.e., chemometrics) is essential for pattern recognition or modelling (see Figure 3 for an overall approach).

As an exploratory technique that reveals underlying patterns in the data, principal component analysis (PCA) is the most widely applied unsupervised method [33]. It explores the relationship between individual observations and reveals the trends, or groups within the multivariate space [58]. PCA is also applied as a dimension reduction technique that explains the variance of the data matrix in terms of principal components, those being a small number of non-dependent factors containing important information from the original set [59]. Other than differentiating among samples and potentially revealing clustering according to region of origin, for example, data compression with PCA can also be useful prior to other statistical treatments [58]. Notably, PCA is used for two-way array data. With three-way data such as EEMs arising from total fluorescence spectroscopy, PARAFAC can be used instead to decompose and extract the information into different components that describe the variability of the EEM data more specifically [47]. These aspects are revealed in Figure 3 as early steps in the overall data analysis process.

For classification purposes, supervised statistical approaches such as discriminant analysis methods are widely applied in authentication of wine (Figure 3). Among spectroscopic studies, PLS-DA and LDA methods have mainly been considered. With LDA (or canonical variate analysis), linear combinations of the original variables (i.e., canonical variates) are estimated that provide maximum separation between classes (groups) while minimising the variance within each class. However, for LDA, the number of training samples needs to be larger than the number of variables, so variable selection by PCA needs to occur with spectroscopic analysis prior to classification with LDA [60]. On the other hand, PLS-DA uses regression to estimate the class of a sample from the variables obtained from a spectral technique, whereby the entire data matrix is regressed on a binary-coded response array and samples are classified according to their predicted values. In their study, Geană et al. showed that LDA works well for classification according to variety with UV–Vis data and PLS-DA improved the classification with FT-IR data [27]. The disadvantage of PLS-DA is that a sample can remain unclassified if it does not belong to any of the pre-defined classes [61].

Another commonly applied supervised technique for classifying wine involves class modelling (Figure 3), and specifically SIMCA, in which similarities among samples belonging to the same class are captured. As explained by Suciu et al., SIMCA is built around PCA and is sensitive enough to identify false outliers to improve the robustness of the model [47]. The advantage of SIMCA over discriminant analyses is that it defines the acceptance area around the target class, which enables delimiting of the target objects from any other objects and classes, and allows assignment of a new sample if it locates in the assigned area of the class [61]. However, due to overlapping of regions, some samples might be classified in one or more classes, and as Rodionova et al. concluded, all classification tasks require the use of an appropriate chemometric approach [61]. That will include the application of new methods, and indeed in more recent years the development of machine learning techniques has shown great potential as they offer advantages in classification compared to conventional methods.

Machine learning started gaining attention in food analysis due to the possibility of performing both linear and non-linear classifications [33]. Among the approaches (Figure 3), SVM has been explored more for wine authentication [62], in conjunction with UV–Vis (Table 1) and NIR (Table 2). SVM is an effective machine learning technique suitable for both classification and regression analysis. It is based on a kernel extension of a binary linear classifier that classifies samples in a hyperplane built according to the features [63]. When a sample set is not balanced over the classes, however, the classification accuracy from SVM may be affected. An alternative machine learning technique involves a decision tree (DT) approach, of which there are several variations, with the most well-known being classification and regression tree (CART). DT methods divide the samples into classes based on the value of certain variables and can be boosted (iterative model) or bagged (independent models including RF), whereby the DT modelling is repeated on random subsets of samples combined into ensembles [64]. Such methods show higher accuracy in classification, are unaffected by outliers or non-linear relationships, and can suitably address class imbalance problems [64]. XGBDA is one such algorithm based on a boosted DT that has recently been applied for the first time (Table 3) to geographical and varietal authentication of red wine using fluorescence spectroscopy [11,48]. Another method for non-linear classification is nearest class mean (NCM), which has rarely been applied in spectroscopic analysis of wine. After dimension reduction of data (from NMR for example, Table 5) with PCA followed by LDA to maximise class separation, NCM can then be used to assign wines to a class with the minimum distance between the respective model class mean and the test-set object [52]. Artificial neural network (ANN) is another option that performs well in classifying samples with non-linear behaviour [65] and has shown acceptable results in variety classification of grapes using FTIR [66]. Although ANN (and CART) has been applied to classification of wine based on anthocyanins [67] or volatiles [68] using chromatographic techniques, there did not appear to be any examples involving spectroscopic data.

### Steps of Chemometric Analysis

It is important to appreciate the key stages in any chemometric approach that need to be followed to complete the process (Figure 3).

Apart from the modelling aspects mentioned in the preceding paragraphs, applying a proper spectral pre-processing method depends on the nature of the data set. Noise reduction and baseline offset are common for all spectroscopic techniques and mainly involve smoothing using techniques like the Savitzky-Golay algorithm [69]. For vibrational spectroscopic data, multiplicative scatter correction and standard normal variate methods are utilised for applying corrections to the spectra by comparing signal intensities to a reference signal. Instead for EEM data, correction of Rayleigh masking, Raman scattering, and IFE corrections need to be used. Other than the analytical artefacts, issues can arise with sample variations. For these, it is important to apply pre-processing methods such as normalisation to remove differences due to dilution and for equalising the integral of peaks of the spectra. Different scaling methods, such as autoscaling, and transformations like mean centring are useful in identifying the important variables among others [69].

Data fusion is another practice that can be carried out to enhance the classification of products and predict their properties. After data pre-processing, data fusion (usually involving variables from complementary techniques) can be carried out in different ways. As a relatively simple approach, low-level data fusion (Figure 3) uses measurements directly from different techniques. In contrast, mid-level fusion uses features obtained from the data sources such as PCA scores, which is important when data is diverse in size or scale. In high-level fusion, the results of the different individual models of the data are combined and applied to the classification problem [65].

Another essential aspect of the chemometric application is model validation (Figure 3). After implementation, a classification model’s validity has to be verified with a validation sample set, to avoid overfitting of the model and to assess its accuracy. It can be categorised as internal validation when separated into calibration and validation sets, and external validation when independent test sets are used. Cross-validation (CV) is the most commonly applied validation method, consisting of different techniques such as leave-one-out CV, multi-fold (*k*-fold)/Venetian blinds CV, contiguous blocks, and random subsets. There can also be split validation, where the whole data set is divided according to different methods such as random, duplex or Kennard-Stone, [64]. In selecting a suitable validation method, it is important to consider the number of samples in the set, otherwise validation can lead to inaccurate results in prediction of new samples. For example, if the sample set is not large enough, CV methods would be more suitable over split analysis [70]. Furthermore when considering the sample size, classification methods have been identified as being less susceptible to sample size variation, such as the RF technique [71]. However, it is difficult to suggest which combination of classification/validation approaches would always give significantly better results than any other [72].

Whichever methods are chosen, performance indicators are important parameters to consider in the model validation process. In authentication applications, misclassification and correct classification rates, expressed as a percentage of all samples in a class or an overall average, are most commonly applied to evaluate the model performance in the studies reviewed in this paper. Other than these measures of accuracy, sensitivity and specificity can also be evaluated as performance indicators [48]. Measures of performance specifically for multivariate regression models include coefficient of determination, which represents the goodness of fit of the model based on the training set, and the root mean square errors of calibration and prediction (RMSEC and RMSEP), which are used to understand the predictive capability of the models [64].

## 4. Future Trends and Directions

Given the international nature of the modern wine trade, reliable methods for assessment of wine authenticity are required to guarantee customer satisfaction of product quality. Potential approaches need to satisfy a number of criteria, foremost of which is having sensitivity to accurately classify non-authentic wines with a high degree of certainty without misclassifying authentic wines as fraudulent. Ideally, a suitable method also needs to be rapid and easily applied, even in a supply chain setting. Therefore, spectroscopic techniques are destined to play a major role due to meeting criteria such as being rapid, user-friendly, and cost-effective.

Among the range of current spectral tools, there have been a number of breakthroughs in the application of spectroscopy for wine analysis. One exciting development is the ability to undertake non-destructive wine measurements through-bottle using various spectroscopic techniques (NIR-Vis, Raman, NMR) which has been successful to a certain extent in identifying oxidation and illegal or hazardous contaminants [73]. Nevertheless, improvements in available techniques or development of new ones to identify chemical markers for geographical, varietal, or vintage authentication is ongoing. NMR provides a powerful platform but is not readily deployable in the production or supply chains, in contrast to things like NIR and UV–Vis. Most recently, great promise has been shown with fluorescence spectroscopy with XGBDA modelling, and indeed, the application of powerful chemometric methods such as machine learning algorithms along with spectroscopic data could be exploited further. Improving the user-friendliness of the statistical techniques is important, however, as that will permit non-specialists to apply them within industry. This could conceivably be solved with the development of cloud-based processing and database management, which could also provide accessibility for authorities for the construction of a robust authenticity database containing rigorous details. Ultimately, integration of innovative technology and modelling approaches will add a new dimension to wine authentication and improve the functionality of the current processes. Importantly, this will give consumers added confidence that the wines they purchase and consume are authentic.

## Figures and Tables

**Table 1:** Examples of UV–visible spectroscopy in combination with chemometrics for wine authentication.

Spectroscopic Technique	Spectral Range	Parameters for Authentication	Classification Method ^1^	Remark	Reference
UV–Vis	200–800 nm	Geographical origin (Spanish denomination of origin)	SVM	Correct classification rates above 96%	[18]
UV–Vis	200–500 nm	Geographical origin of Argentinian regions	PCA, LDA, PLS-DA	Correct classification with LDA and PLS-DA methods of 100%	[19]
UV–Vis	300–800 nm	Discrimination by origin, grape variety and ageing process	PCA, SIMCA	Correct classification of 90% for geographical origin, and 75% for variety and ageing process	[20]
UV–Vis	240–700 nm	Discrimination according to grape variety, ageing process and barrel/container type	OPLS-DA	Correct classification of 97% for variety, 73% for ageing process and 100% for container type	[21]

^1^ SVM, support vector machine; PCA, principal component analysis; LDA, linear discriminant analysis; PLS-DA, partial least squares-discriminant analysis; SIMCA, soft independent modelling of class analogy; OPLS-DA, orthogonal projections to latent structures discriminant analysis.

**Table 2:** Examples of IR spectroscopy in combination with chemometrics for wine authentication.

Spectroscopic Technique	Spectral Range	Parameters for Authentication	Classification Method ^1^	Remark	Reference
MIR	5012–926 cm^−1^	Discrimination of red and white varieties from Australian regions	PCA, LDA	Correct classification of red varieties, 96% and white varieties, 94%	[26]
UV–Vis, NIR and MIR	400–2500 nm (UV–Vis and NIR) and 4000–400 cm^−1^ (MIR)	Geographical origin of Sauvignon blanc wines from Australia and New Zealand	PCA, SIMCA, PLS-DA	Correct classification using PLS-DA with: UV–Vis, 67%; NIR, 76%; MIR, 90%; and combined IR spectra, 93%	[23]
UV–Vis/NIR	190–2500 nm	Discrimination of white wines (Albariño cultivar) from Rías Baixas subzones in Spain	PCA, LDA, SIMCA, SVM	Correct classification using: LDA, 86%; SIMCA, 56%; and SVM, 84%	[30]
NIR and MIR	1750–1000 cm^−1^ and 4555–4353 cm^−1^	Geographical origin of Cabernet Sauvignon wines from Australia, Chile, and China	PCA, SIMCA, DA	Correct classification using: SIMCA, 97%, 97%, and 92% for Australian, Chilean, and Chinese wines; and DA, 86%, 85%, and 77%, respectively.	[31]

^1^ PCA, principal component analysis; LDA, linear discriminant analysis; SIMCA, soft independent modelling of class analogy; PLS-DA, partial least squares-discriminant analysis; SVM, support vector machine.

**Table 3:** Examples of Raman spectroscopy in combination with chemometrics for wine authentication.

Spectroscopic Technique	Spectral Region	Parameters for Authentication	Classification Method ^1^	Remark	Reference
FT-Raman	1700–0 cm^−1^ (Stokes), −1000–0 cm^−1^ (anti-Stokes) (laser emitting at 1064 nm)	Discrimination of wines geographically, varietally, and by vintage	LDA	Correct classification of: variety, 84%; geographical origin, 100%; and vintage, 95%	[37]
SERS	3350–200 cm^−1^ (laser emitting at 532 nm)	Discrimination of wines geographically (Romanian and French and different Romanian regions), varietally, and by vintage	LDA	Correct classification of: variety, 90%; geographical origin, 83% among Romanian wines and 100% between countries; and vintage, 90%	[35]
SERS	1600–450 cm^−1^ (laser emitting at 785 nm)	Discrimination of wines according to variety and producer	PCA, SIMCA	Correct classification of: variety, 87%; and producer, 93%	[38]

^1^ LDA, linear discriminant analysis; PCA, principal component analysis; SIMCA, soft independent modelling of class analogy.

**Table 4:** Examples of fluorescence spectroscopy in combination with chemometrics for wine authentication.

**Spectroscopic Technique**	**Spectral Region**	**Parameters for Authentication**	**Classification** **Method ^1^**	**Remark**	**Reference**
Synchronous fluorescence spectroscopy	λ_ex_ = 250–350 nm and λ_em_ = 250–500 nm	Discrimination of white wines according to variety in Tokaj (Slovakia)	PCA, LDA	Correct classification of variety, 100%	[46]
Total fluorescence spectroscopy	EEM λ_ex_ =240–800 nm and λ_em_ 242–824 nm	Discrimination of Cabernet Sauvignon wines from Australia and Bordeaux, France	SVMDA XGBDA	Correct classification of geographical origin using: XGBDA, 100%; and SVMDA, 85%	[11]
Total fluorescence spectroscopy	EEM λ_ex_ = 250–500 nm and λ_em_ 275–600 nm	Discrimination of white wine from Romania and France for geographical origin and variety	PARAFAC, SIMCA	Correct classification of: variety, 97%; and geographical origin, 98%	[47]
Total fluorescence spectroscopy	EEM λ_ex_ =240–700 nm and λ_em_ 242–824 nm	Discrimination of red wine varieties from different Australian regions for variety and geographical origin	XGBDA	Correct classification of: variety, 100%; and geographical origin, 99.7%	[48]

^1^ PCA, principal component analysis; LDA, linear discriminant analysis; SVMDA, support vector machine discriminant analysis; XGBDA, extreme gradient boosting discriminant analysis; PARAFAC, parallel factor analysis; SIMCA, soft independent modelling of class analogy.

**Table 5:** Examples of NMR spectroscopy in combination with chemometrics for wine authentication.

Spectroscopic Technique	Spectral Range	Parameters for Authentication	Classification Method ^1^	Remark	Reference
^1^H NMR	0.5–9.5 ppm	Discrimination of wines geographically (German wine regions), varietally, and by vintage	PCA, LDA, NCM	Correct classification of: variety, 95%; geographical origin, 89%; and vintage, 96–97%	[52]
^1^H NMR	0.8–9.7 ppm	Varietal differentiation of red and white wines produced in different regions in China	PCA, LDA	Correct classification of: red wines, 83%; and white wines, 94%	[53]
^1^H NMR	0.0–10.0 ppm	Varietal differentiation of red and white wines produced in Czech Republic	PCA, RF	Correct classification of: most varieties, ~70%; and type of wine, 92%	[54]

^1^ PCA, principal component analysis; LDA, linear discriminant analysis; NCM, nearest class mean; RF, random forest.

**Table 6:** Summary of spectroscopic techniques applied to wine authentication [33,57].

Technique	Chemical Marker	Advantages	Disadvantages
UV–Vis	Hydroxybenzoic acids, hydroxycinnamic acids, flavan-3-ols, flavonols, and anthocyanin glucosides	Simple analysis, low cost, small volume	Difficulty in identifying specific analytes
IR	Organic acids, alcohols, reducing sugars, and polyphenols	Rapid, simple, qualitative and quantitative analysis	Sensitive to pH and temperature, high interference of water (NIR)
Raman	Organic acids, alcohols, sugars, phenolics	Rapid, small volume, low impact of water	Weak signals, extensive pre-processing requirements
Fluorescence	Phenolics, pigments, vitamins, amino acids	Rapid, sensitive and selective, qualitative and quantitative analysis	Extensive pre-processing requirements, quenching effect
NMR	Phenolics, alcohols, organic acids, amino acids, sugars	Rapid, selective, repeatable and reproducible	Costly equipment, experienced analyst required
